# Left ventricular global longitudinal strain in patients treated with immune checkpoint inhibitors

**DOI:** 10.3389/fonc.2024.1453721

**Published:** 2024-12-24

**Authors:** Ece Celebi Coskun, Alper Coskun, Ahmet Bilgehan Sahin, Fatih Levent, Eyup Coban, Fatih Koca, Seda Sali, Omer Furkan Demir, Adem Deligonul, Erhan Tenekecioglu, Erdem Cubukcu, Turkkan Evrensel, Fahriye Vatansever Agca

**Affiliations:** ^1^ Department of Cardiology, University of Health Sciences Bursa Yuksek Ihtisas Training and Research Hospital, Bursa, Türkiye; ^2^ Department of Medical Oncology, Bursa Uludag University School of Medicine, Bursa, Türkiye; ^3^ Department of Medical Oncology, University of Health Sciences Bursa City Hospital, Bursa, Türkiye

**Keywords:** GLS, global longitudinal strain, immunotherapy, cancer, cardiotoxicity, myocarditis

## Abstract

**Background:**

Immune checkpoint inhibitors (ICI) are generally associated with rare cardiac side effects, yet instances like myocarditis can be fatal. Therefore, detecting and managing left ventricular dysfunction early in ICI therapy is vital.

**Objectives:**

This study aims to evaluate whether left ventricular global longitudinal strain (LV GLS) is a predictor for early detection of cardiac dysfunction in patients receving ICI.

**Methods:**

This retrospective cohort study included 44 cancer patients who received ICI therapy and underwent pre- and post- treatment assessments of left ventricular ejection fraction (LVEF) and LV GLS between May 2022 and November 2023. Retrospective comparisons and evaluations were conducted on pre-treatment and 3-month interval LVEF and LV GLS measurements during the first year of treatment.

**Results:**

The median follow-up duration was 5.3 months (0.5-18.9). No statistically significant difference between baseline and subsequent time points was observed in LVEF and LV GLS values (p>0.05). At the 3-month evaluation, a notable decrease in LVEF and LV GLS was observed in two patients. One patient with reduced LVEF and LV GLS succumbed to myocarditis, and another experienced sudden death of unknown etiology. The other two patients had decreased LV GLS with normal LVEF. Subsequent follow-ups of the patients exhibiting decreased LV GLS alone revealed no further decline in LVEF or LV GLS.

**Conclusion:**

In our study, a reduction in LV GLS did not demonstrate a significant role in the early prediction of ICI-related myocarditis or cardiac dysfunction. Further validation through multicenter, large-scale, prospective studies with extended follow-up periods is needed to confirm these findings.

## Introduction

Immunotherapy has emerged as a significant treatment modality, either alone or in combination with chemotherapy, radiotherapy, and targeted therapies for various cancer types, notably malignant melanoma and renal cell carcinoma. Its utilization has significantly enhanced treatment response rates and overall survival, thus solidifying its role in clinical practice over the past two decades. Immune checkpoint inhibitors (ICI), a form of immunotherapy, function by stimulating the immune system through the inhibition of immune checkpoint proteins such as lymphocyte activating gene-3, programmed cell death-1 (PD-1), and cytotoxic T lymphocyte antigen-4 (CTLA-4), which are integral components of the T lymphocyte negative costimulation pathway (1). Agents such as ipilimumab and tremelimumab target CTLA-4, while nivolumab, pembrolizumab, cemiplimab, dostarlimab, atezolizumab, avelumab, and durvalumab act on the PD-1 pathway (2).

Unlike conventional systemic chemotherapy and radiotherapy, ICI therapy is associated with distinct side effects. Approximately 65% of patients treated with ICI experience systemic side effects, primarily affecting the thyroid, colon, liver, skin, and heart, with around 13-23% classified as grade 3 or 4 (3, 4). Immune-related adverse events predominantly impact the gastrointestinal, endocrine, and dermatological systems, with less frequent but potentially fatal occurrences affecting the neurological, cardiac, and pulmonary systems (5). Although cardiovascular complications are less common with immunotherapy, they often carry a poor prognosis. These include myocarditis, pericarditis, Takotsubo cardiomyopathy, vasculitis, venous thromboembolism, arrhythmias (supraventricular and ventricular), and acute coronary syndromes. While most adverse events occur within the first three months, the literature suggests these complications can manifest between 2 and 454 days after initiation of the treatment (6). Although the most prevalent ICI-related cardiac manifestation is electrocardiographic changes, myocarditis is the most significant cardiovascular toxicity in clinical practice. ICI-associated myocarditis occurs in 1-2% of patients, with mortality rates ranging from 25-50%, often accompanied by clinical myositis and/or myasthenia gravis in 25% of cases (7, 8).

High-risk patients for ICI-related cardiac complications consist of those receiving combination ICI therapy or ICI with other cardiotoxic agents, individuals with a history of cardiovascular disease, and patients developing cardiac dysfunction following previous cancer treatments. The European Society of Cardiology (ESC) recommends baseline echocardiography in all high-risk patients and regular cardiovascular assessments at 6 to 12-month intervals for those undergoing long-term ICI therapy. Moreover, all patients receiving ICI treatment should undergo baseline cardiovascular evaluation, including electrocardiography (ECG), troponin, and pro-brain natriuretic peptide (pro-BNP), with echocardiography recommended even for low-risk patients (9). ESC Cardio-Oncology guidelines advocate including left ventricular global longitudinal strain (LV GLS) examination alongside conventional cardiac assessments, ECG, echocardiography, troponin, and pro-BNP measurements in patients with suspected cardiovascular complications (9). LV GLS, derived from 2-dimensional echocardiography images acquired from the apical 2-3-4 chambers, is a predictive indicator for the early detection of asymptomatic cardiac dysfunction in patients undergoing chemotherapy (10). A study involving 1329 healthy individuals established the reference range for GLS as -24% to -16% (11). The World Alliance Societies of Echocardiography (WASE) have endorsed normal GLS values ranging from -24% to -17% in males and -26% to -18% in females (12). Furthermore, a meta-analysis encompassing 16 studies comprising 5721 cases concluded that, compared to left ventricular ejection fraction (LVEF), LV GLS emerges as a more robust predictor of adverse outcomes, including all-cause mortality, cardiovascular mortality, heart failure-related hospitalizations, and malignant arrhythmia development (13).

Despite extensive research on LV GLS concerning chemotherapy and targeted therapies, limited studies focus specifically on patients receiving ICI. Thus, addressing this gap, this study aims to assess the sensitivity and reliability of LV GLS compared to LVEF in the early detection of cancer treatment-related cardiac dysfunction (CTRCD) and to evaluate ICI-associated side effects and cardiovascular surveillance.

## Materials and methods

**Figure 1:**
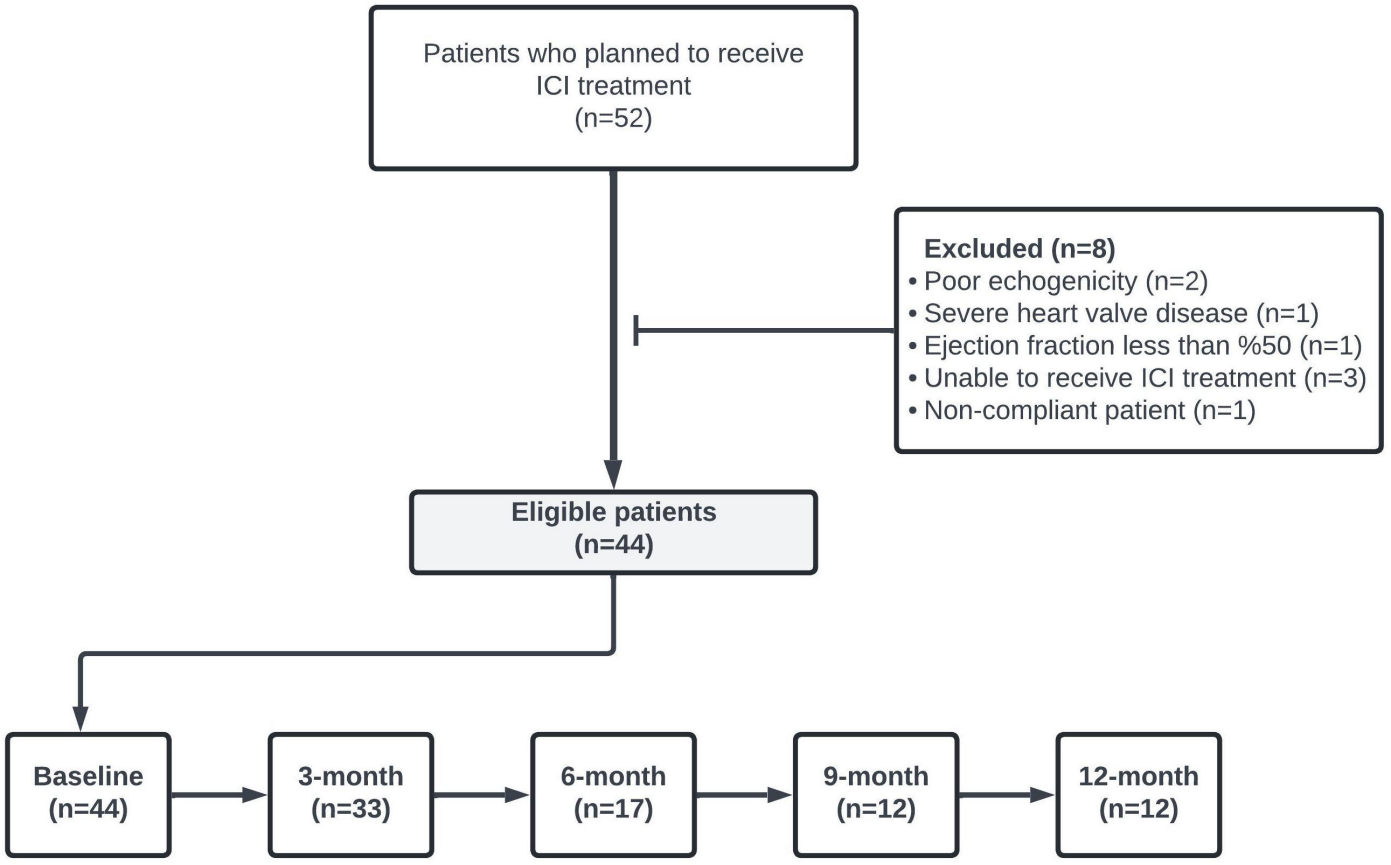
Flow chart of study design.

This clinical observational study evaluated all patients aged ≥18 years who admitted to the Cardiology Clinic of the University of Health Sciences Bursa Yuksek Ihtisas Training and Research Hospital between May 1, 2022, and November 1, 2023 and initiated ICI for cancer management. Patients’ data were retrospectively examined. A total of 44 patients who underwent LVEF and LV GLS measurements at least once before ICI treatment and met the study inclusion criteria were included in the analysis. The study’s flowchart is depicted in **Figure 1**. Patients with cardiovascular interventions due to coronary artery disease within three months, LVEF less than 50%, poor echogenicity on echocardiography, cardiomyopathy, severe valvular heart disease, impaired renal function (GFR<30 ml/min), and severe hepatic failure were excluded.

Data pertaining to the patients’ characteristics, the ICI treatments, the number of ICI cycles, follow-up durations, and survival status were retrieved from patients’ records and electronic files. Transthoracic echocardiography measurements were performed before treatment, and the 3-month follow-up examinations in the first year of immunotherapy were retrospectively evaluated. LVEF and LV GLS measurements in transthoracic echocardiography were performed using GE Vivid E95 EchoPac software in the echocardiography laboratory. Left ventricle measurements were acquired using 2-dimensional echocardiography, color, continuous, and pulse Doppler. LV measurements were obtained from apical 4-chamber, apical 2-chamber, and short-axis windows using 3-dimensional echocardiography. By delineating the contours of the endocardial surface of the left ventricle, the software automatically computed motion and analyzed characteristics.

In this study, CTRCD was defined as a decrease below 50% or an increase of 10% or more compared to the baseline of LVEF and a reduction of 15% or more compared to the baseline of LV GLS.

### Ethical approval

Approval for this study was obtained from the Ethics Committee of the University of Health Sciences Bursa Yuksek Ihtisas Training and Research Hospital (decision no: 2011-KAEK-25 2023/11-01). Since our study is a retrospective study, the ethics committee did not request an additional informed consent for the study, apart from the informed consent obtained for the ICI treatment.

### Statistical analysis

The data obtained in the study were statistically analyzed using IBM SPSS version 25.0 software. Categorical variables were presented as number (n) and percentage (%), while continuous variables were expressed as mean ± standard deviation (SD), median, minimum, and maximum values. The normal distribution of the data was assessed using the Kolmogorov-Smirnov test. For variables demonstrating normal distribution, the Dependent Samples t-test was employed as the paired repeated measurement test, whereas for variables not displaying normal distribution, the Wilcoxon test was utilized. A significance level of p < 0.05 was considered statistically significant.

## Results

The general characteristics of the patients are summarized in **Table 1**. Of the 44 patients, 28 (64%) were male and 16 (36%) were female, with a median age of 64 years (range, 27-82 years) and a median follow-up period of 5.3 months (range, 0.5-18.9 months). Hypertension was the most frequent comorbidity (39%), followed by ischemic heart disease (20%). The most common indication for ICI treatment was non-small cell lung cancer (n=23, 52%). Nivolumab was the most frequently used ICI, administered to 40 (91%) patients. The median number of ICI cycles administered was 7 (range, 1-31).

**Table 1:** The general characteristics of the patients.

Characteristics (N=44)	n (%)
Age, median (minimum-maximum)	64 (27-82)
Gender	
- Female	16 (36.4%)
- Male	28 (63.6%)
Comorbidity	
- Hypertension	17 (38.6%)
- Diabetes Mellitus	4 (9.1%)
- Ischemic Heart Disease	9 (20.5%)
Smoking Status	29 (65.9%)
Smoking (package/year), mean ± SD	38.7 ± 19.0
Pathological Type	
- Non-Small Cell Lung Cancer	23 (52.3%)
- Malignant Melanoma	15 (34.1%)
- RCC	2 (4.5%)
- Bladder	2 (4.5%)
- Skin SCC	1 (2.3%)
- Esophagus	1 (2.3%)
Type of ICI	
- Nivolumab	38 (86.4%)
- Pembrolizumab	2 (4.5%)
- Ipilimumab	1 (2.3%)
- Nivolumab + Ipilimumab	1 (2.3%)
- Nivolumab + Cabozantinib	1 (2.3%)
- Nivolumab + Carboplatin + Pemetrexed	1 (2.3%)
ICI Treatment Setting	
- Metastatic	39 (88.6%)
- Adjuvant	4 (9.1%)
- Neoadjuvant	1 (2.3%)
ICI Cycles, median (minimum-maximum)	7 (1-31)

LVEF and LV GLS measurements were conducted via transthoracic echocardiography in 44 patients at baseline, 33 at the 3rd month, 17 at the 6th month, 12 at the 9th month, and 12 at the 12th month. LVEF and LV GLS measurements are presented in **Table 2**. With the LV GLS reference value set at -17%, it was determined that 28 patients (%64) presented with decreased LV GLS at baseline. No statistically significant difference between baseline and subsequent time points was observed in LVEF and LV GLS values (p>0.05) (**Table 3**).

**Table 2:** The left ventricular ejection fraction (LVEF) and left ventricular global longitudinal strain (LV GLS) values of patients before and during immune checkpoint inhibitors treatment.

	Baseline (n=44)	3-month (n=33)	6-month (n=17)	9-month (n=12)	12-month (n=12)
**LVEF % (median, minimum-maximum)**	60.0 (48.0-65.0)	60.0 (45.0-60.0)	60.0 (60.0-60.0)	60.0 (60.0-60.0)	60.0 (60.0-60.0)
**LV GLS (mean ± standard deviation)**	-16.0 ± 0.4	-16.0 ± 0.5	-16.5 ± 0.5	-16.2 ± 0.8	-16.1 ± 0.7

**Table 3:** Comparison of patients left ventricular ejection fraction (EF) and left ventricular global longitudinal strain (GLS) measurements before and during immune checkpoint inhibitors treatment.

	3-month EF	6-month EF	9-month EF	12-month EF	
**Baseline EF**	0.50*	0.25*	1.00*	1.00*	p-value

	3-month GLS	6-month GLS	9-month GLS	12-month GLS	
**Baseline GLS**	0.45**	0.88*	0.45*	0.24*	p-value

*Wilcoxon test, **Paired samples t-test.

At the 3-month evaluation, a significant decrease in both LVEF and LV GLS was observed in 2 (5%) patients, while a reduction in only LV GLS was noted in two (5%) patients. One of the patients with decreased LVEF succumbed to myocarditis shortly after the cardiac assessment (Grade 5 myocarditis), while the other patient experienced sudden death. ICI treatments were continued in the two patients with decreased LV GLS but normal LVEF. Subsequent follow-up examinations revealed stable LV GLS levels and no decline in LVEF. Grade 2 pericarditis developed in one (2%) patient during the 3-month ICI treatment, but treatment was continued without any complications. At the 12-month follow-up, although pericardial effusion persisted, there was no decrease in LVEF or LV GLS. Detailed data regarding these five patients’ characteristics and clinical follow-up findings are outlined in **Table 4**.

**Table 4:** General characteristics and clinical follow-up of patients with cardiac events.

	Cancer Type	ICI agent	LV GLS					Follow-up
			Baseline	3-month	6-month	9-month	12- month	
Case 1	Malignant Melanoma	Nivolumab	-14,1	-10,2	–	–	–	Grade 5 myocarditis
Case 2	NSCLC	Nivolumab	-14,8	-10,4	–	–	–	Sudden cardiac death
Case 3	Malignant Melanoma	Pembrolizumab	-19	-16,1	-16,9	-16,5	-16,1	ICI treatment continues
Case 4	NSCLC	Nivolumab	-18	-12,7	-12,5	–	–	ICI treatment continues
Case 5	NSCLC	Nivolumab	-16	-16,2	-16,1	-14,8	-15,4	Grade 2 pericarditis

When considering the LV GLS reference value as -17%, it was observed that 20 out of the 33 patients who underwent a 3-month LV GLS evaluation had initially reduced LV GLS. In contrast, 13 patients had initially preserved LV GLS. In 17 out of 20 patients with initially reduced GLS, ICIs were used as a second-line or later therapy in a metastatic setting. These patients had received prior systemic treatment (mono/doublet chemotherapy, tyrosine kinase inhibitors) before ICI therapy. The other three patients had a pre-existing coronary artery disease. In our study, LV GLS in four patients significantly decreased during ICI treatment. Three of them had initially decreased LV GLS and one had preserved LV GLS. The three patients with initially reduced GLS had received other anticancer treatments prior to ICI therapy. The patient with initially preserved GLS, however, was one who had not received prior anti-cancer treatment and was receiving adjuvant ICI therapy. The rate of substantial reduction of LV GLS in patients with initially preserved LV GLS was 7.6%, while it was 15% in those with reduced LV GLS.

## Discussion

As observed in reports studying the cardiotoxicity of cytotoxic chemotherapy and targeted agents, a decline in LV GLS in patients undergoing ICI therapy may serve as a significant predictive marker for early detection of cardiac dysfunction. From this perspective, our study investigated the predictive value of LV GLS in anticipating cardiac toxicity in patients receiving ICI treatment. However, upon comparing LV GLS values at baseline and subsequent time points, no statistically significant decrease in LV GLS was detected.

Previous investigations in patients receiving anthracycline-based chemotherapy and/or targeted treatments, such as trastuzumab, for cancer management have demonstrated LV GLS as a significant predictor for early detection of CTRCD, surpassing LVEF in sensitivity for early cardiovascular toxicity (10, 14–16). To our knowledge, limited literature exists regarding evaluating LV GLS in patients treated with ICI (17–19).

The study conducted by Awadalla et al. was robust due to both the large number of patients and the high rate of patients developing ICI-related myocarditis. This study demonstrated a significant reduction in LV GLS in patients who developed ICI-related myocarditis. Furthermore, it was found that decreased GLS has a strong relationship with major adverse cardiac events (MACE) independent of LVEF. Additionally, this study indicates that GLS assessment in all patients receiving ICI therapy is of limited efficacy, as myocarditis related to ICI has a low prevalence compared to the overall evaluation of all myocarditis cases (17). In another study evaluating patients receiving ICI treatment, a significant relationship was shown between elevated high-sensitivity troponin I (hsTnI) and reduced GLS. In this study, similar to ours, a reduction in GLS was observed in ICI-related myocarditis patients; however, the reduction in GLS could not be shown to contribute to the early detection of ICI-related myocarditis (18).

In the study conducted by Li et al. on patients with ICI-related myocarditis using cardiac magnetic resonance imaging (CMR), when comparing corticosteroid-sensitive and corticosteroid-resistant patients with a control group, it was demonstrated that the significant decrease in subendocardial GLS could serve as a useful parameter for the early diagnosis of corticosteroid-resistant ICI-related myocarditis (19). Current ESC cardio-oncology guidelines recommend CMR evaluations in addition to LV GLS and other routine cardiovascular assessments when myocarditis is suspected in high-risk patients (9).

The mechanism underlying ICI-related cardiotoxicity remains unclear. However, it is commonly theorized to involve an autoimmune response resulting from T cell over-activation with ICI, along with the presence of homologous antigens in both heart cells (cardiomyocytes) and tumor cells (7). Studies have demonstrated T cell clonal expansion in both tumor cells and cardiomyocytes in patients receiving ICI therapy. Programmed death ligand-1 (PDL-1) expression in human cardiomyocytes is suggested to increase myocardial damage, with experimental rat models showing elevated PD-1 and PDL-1 levels following myocardial insult (20). While the binding of PDL-1 to PD-1 typically serves as a protective mechanism against autoimmune myocarditis, ICI use inhibits this binding, thereby increasing the risk of cardiac damage (21). In this context, the assessment of LV GLS is believed to provide valuable insights into the early diagnosis and management of ICI-related myocarditis. Nevertheless, further large-scale prospective studies are warranted to validate these findings.

In routine oncology practice, ICI-related cardiotoxicity is primarily associated with myocarditis, which carries significant clinical implications due to mortality rates of up to 50%. Consistent with existing literature, myocarditis was observed in one (2.2%) patient in the third month of treatment, shortly after cardiovascular evaluation. Despite aggressive treatment, including high-dose steroids, plasmapheresis, and supportive measures, the patient succumbed.

Notably, this patient exhibited a baseline LV GLS below -17%, and despite being asymptomatic at the 3-month examination, experienced a significant decrease in both LVEF and LV GLS.

Our study observed that patients with reduced LV GLS had a significant decrease of 15%, while those with preserved LV GLS had a decrease of 7.6%. A study of 188 cancer patients undergoing anthracycline-based chemotherapy found that CTRCD was more common in those with initially reduced LV GLS compared to those with preserved LV GLS (22). In this study, 33% of the patients were male, and the reference value for LV GLS was -18%. However, in our study, as 64% of the patients were male, we accepted the reference value of LV GLS as -17%, adjusting for gender according to the reference values determined by WASE (12).

Limitations of this study include the relatively small sample size and short follow-up period, primarily attributed to the advanced disease stage and frequent disease progression and mortality in patients receiving ICI therapy. Additionally, the study did not include data regarding ECG changes and arrhythmias, which are pertinent to ICI-related cardiotoxicity. One of the key limitations of this study is the absence of cardiac biomarker monitoring both at baseline and during the course of ICI therapy.

In conclusion, LV GLS is identified as a sensitive and reliable indicator for the early detection of CTRCD. Although our study did not show a statistically significant difference due to the limited number of patients. To validate these findings, it is necessary to conduct multicenter, large-scale, prospective studies with longer follow-up periods.

## Data Availability

The original contributions presented in the study are included in the article/supplementary material. Further inquiries can be directed to the corresponding author.
